# Prevalence of Celiac Disease in Children and Adolescents With Type 1 Diabetes in Saudi Arabia: A 20‐Year Experience in a Tertiary Care Center

**DOI:** 10.1155/ije/5921264

**Published:** 2026-11-09

**Authors:** Adnan AlShaikh, Abdulaziz Alghamdi, Sahl Alshaikh, Yahya Almatihmy, Mohammed Hasosah, Shaima Alghamdi, Aljowharah AlSehaibany, Leena Waqar, Joud Alshaikh, Mohamed Ahmed, Ashraf Soliman

**Affiliations:** ^1^ King Abdulaziz Medical City National Guard Health Affairs Jeddah Saudi Arabia mngha.med.sa; ^2^ King Saud Bin Abdulaziz University for Health Sciences Ministry of National Guard Health Affairs Jeddah Saudi Arabia mngha.med.sa; ^3^ King Abdullah International Medical Research Center Jeddah Saudi Arabia kaimrc.med.sa; ^4^ King Abdulaziz University Jeddah Saudi Arabia kau.edu.sa; ^5^ Department of Paediatrics Hamad Medical Centre Doha Qatar

**Keywords:** autoimmune thyroiditis, celiac disease, diabetic ketoacidosis, duodenal biopsy, endoscopy, HbA1c, hypoglycemia, serologic tests, Type 1 diabetes mellitus

## Abstract

Variable rates of co‐occurrence of celiac disease (CD) and Type 1 diabetes mellitus (T1DM) in the pediatric population have been reported across numerous studies. This retrospective study aimed to determine the prevalence of CD and other autoimmune disorders in children and adolescents (1 to 18 years) from Saudi Arabia with T1DM at King Abdulaziz Medical City‐Jeddah. Additionally, we assessed the specificity and sensitivity of different CD serological diagnostic tests. A total of 595 children and adolescents with T1DM were included in the study. Positive CD serological tests were reported in 9.4% and positive biopsy in 5.4%. Duodenal biopsy confirmed CD in 62.5% of children and adolescents with positive tissue transglutaminase immunoglobulin A (IgA), 65.6% with positive endomysia antibodies, and 46.9% with positive anti‐gliadin antibodies. The tissue transglutaminase IgA test at diagnosis demonstrated the highest specificity (91%), while the anti‐gliadin antibody IgA test exhibited the highest sensitivity (68.2%). Children and adolescents with diabetes and CD exhibited a significantly higher prevalence of Hashimoto’s thyroiditis (18.8%) compared to those without CD (8.3%). No significant differences were observed in anthropometric parameters between the two groups. However, children and adolescents with both diseases demonstrated higher hemoglobin A1c levels and more frequent occurrence of episodes of ketoacidosis and hypoglycemia. These glycemic abnormalities may be attributed to the carbohydrate content of gluten‐free (GF) foods and/or poor compliance with GF diets, leading to nutrient malabsorption. A precise diagnosis of CD through serological testing, selected endoscopic evaluation, and dietary control, including accurate carbohydrate counting in GF foods along with adjustments in prandial insulin doses, can significantly enhance glycemic control in these patients.

## 1. Introduction

Type 1 diabetes mellitus (T1DM) is an autoimmune disorder characterized by immune‐mediated destruction of pancreatic islet *β*‐cells, leading to insulin deficiency [1]. The condition commonly manifests in childhood and adolescence, posing a substantial burden on affected individuals and their families [2]. In 2024, there were 46,000 children and adolescents under 20 years of age living with T1DM in Saudi Arabia [3], highlighting the considerable public health impact of this disorder.

Similarly, celiac disease (CD) is another prominent autoimmune condition, primarily affecting the small intestine’s mucosal lining and resulting from the consumption of gluten. Both CD and T1DM typically onset in early childhood, resulting in a complex interplay of autoimmune phenomena during critical developmental periods [4, 5]. These conditions share a bidirectional relationship, where the presence of one increases the risk of developing the other [6, 7]. Recent studies have reinforced this association, demonstrating common genetic and environmental factors contributing to their coexistence [8].

The prevalence of CD among children with T1DM has been extensively studied, with a meta‐analysis study revealing a global prevalence ranging from 1.1% to 16.6% [9]. In Saudi Arabia, the prevalence is notably elevated compared to the general population, with reported prevalence estimates ranging from 11.5% to 21.2% [10–15]. The high frequency of this co‐occurrence necessitates a comprehensive understanding of its clinical implications in order to optimize treatment and management strategies.

T1DM is also frequently associated with other autoimmune disorders, such as autoimmune thyroiditis and autoimmune adrenal insufficiency [16, 17]. Autoimmune thyroid diseases have been reported to occur in approximately 17%–30% of individuals with T1DM [16]. Children are more likely to present with an autoimmune disorder at the time of diagnosis of T1DM [17]. The presence of these additional autoimmune conditions can complicate the clinical course of T1DM, underscoring the need for an integrated approach to care.

Global data from 65,102 patients with T1DM across 40 countries suggest that CD prevalence in individuals with T1DM is approximately 9%, with Saudi Arabia demonstrating one of the highest levels at 11% [18]. In a cross‐sectional retrospective study, Aljulifi et al. reported a prevalence of CD of 11.5% among adolescents and adults with T1DM [19]. The coexistence of CD and T1DM poses unique challenges to glycemic control, increasing the risk of complications like hypoglycemia and diabetic ketoacidosis (DKA) [20, 21]. This association goes beyond a simple comorbidity relationship and includes a common background of human leukocyte antigen (HLA) predisposition, including HLA‐DR3‐DQ2 and HLA‐DR4‐DQ8 haplotypes, as well as other genetic and environmental factors [22]. Notably, HLA‐DQ8 is detected more frequently than HLA‐DQ2 in patients with CD and T1DM [23]. Poor glycemic control is associated with higher serum anti‐tissue transglutaminase immunoglobulin A (TTG‐IgA) levels and greater damage to the intestinal mucosa in children or adolescents with T1DM at the time of CD diagnosis [24]. Patients with both conditions often experience fluctuations in blood glucose levels, partly due to the dietary restrictions imposed by a gluten‐free (GF) diet and potential nutrient malabsorption. Recent research suggests that tailored dietary interventions and regular monitoring can mitigate these risks and improve clinical outcomes [25, 26].

Understanding the intersection of these autoimmune diseases is critical for developing effective management strategies that address the specific needs of this population. This study aimed to determine the prevalence of CD in children and adolescents with T1DM. Further aims included investigating how CD influences glycemic control in children and adolescents with diabetes, exploring the prevalence of other autoimmune diseases in children and adolescents with T1DM, and evaluating the correlation and effectiveness of serological tests and duodenal biopsy in diagnosing CD in children and adolescents with diabetes.

## 2. Methods

### 2.1. Study Design and Setting

This retrospective cohort study was conducted at the outpatient diabetes clinic at King Abdulaziz Medical City‐Jeddah (KAMC‐J). Data were collected retrospectively from the electronic medical record database (Bestcare EMR) at KAMC‐J for the period between 1 January 2000 and 30 December 2021.

### 2.2. Study Population

The study included all patients aged 1 to 18 years diagnosed with T1DM who attended the outpatient diabetes clinic at KAMC‐J between 1 January 2000 and 30 December 2021. Patients with a confirmed diagnosis of T1DM based on clinical evaluation, biochemical tests, and medical history were included. Individuals with Type 2 diabetes mellitus, secondary diabetes resulting from pancreatectomy, steroid use, or other drug‐induced causes, and individuals with monogenic diabetes were excluded. Patients with incomplete medical records or those who did not undergo serological testing for CD were excluded from the analysis.

### 2.3. Variables

A standardized data collection sheet was used to extract relevant information, including demographic data, clinical history, laboratory results, and outcomes. Demographic and clinical variables were age, sex, duration of diabetes, height, weight, blood pressure, hemoglobin A1c (HbA1c), vitamin D deficiency, type of diabetes therapy, total daily dose, frequency and severity of hypoglycemia, frequency of DKA, and presence of other autoimmune diseases (Hashimoto’s thyroiditis, vitiligo, and adrenal insufficiency).

Type of diabetes therapy was classified as either intensive or conventional. Intensive diabetes therapy was defined as a treatment approach that aims for near‐normal blood glucose levels through frequent monitoring and multiple daily insulin injections or continuous infusion, with dose adjustments based on meals, physical activity, and glucose readings. In contrast, conventional diabetes therapy involves fixed insulin regimens with less frequent monitoring, aiming primarily to prevent symptoms of hyperglycemia without strict glycemic targets. Hypoglycemia severity was categorized as mild (level 1; blood glucose < 70 mg/dL [3.9 mmol/L] but ≥ 54 mg/dL [3.0 mmol/L]), moderate (level 2; blood glucose < 54 mg/dL), and severe (level 3; involves any hypoglycemic episode requiring external assistance due to impaired consciousness or physical function, regardless of the measured glucose level). 25‐Hydroxyvitamin D was measured by Chemiluminescent Microparticle Immunoassay. Blood glucose and HbA1c were measured using the Abbott ARCHITECT c8000. Results of serological tests for CD, including TTG‐IgA, endomysial antibodies IgA (EMA‐IgA), and anti‐gliadin antibodies IgA (AGA‐IgA), were also collected. Results of duodenal biopsies were also collected; biopsies were obtained from the duodenum, including two biopsies from the duodenal bulb, and placed in formalin. Histopathological findings were assessed using Marsh, a classification system used to describe the stages of damage and histological changes in the small intestine observed under a microscope and to diagnose CD.

The diagnosis of CD was based on positive serological tests and confirmation by duodenal biopsy, Serological testing included TTG‐IgA which was measured using Enzyme‐Linked Immunosorbent Assay (ELISA); results were classified as negative (< 4 IU/mL), weak positive (4–10 IU/mL), and moderate to strong positive (> 10 IU/mL). TTG‐IgA is the most sensitive and effective method of CD testing. EMA was assessed using immunofluorescence and categorized similarly to TTG. AGA was evaluated using ELISA, with results interpreted as negative (< 20 IU/mL), weak positive (20–30 IU/mL), and moderate to strong positive (> 30 IU/mL). The diagnosis was made according to the European Society Paediatric Gastroenterology, Hepatology and Nutrition guidelines for diagnosing Celiac Disease [27]. At KAMC‐J, periodic screening for CD was performed in line with the International Society for Paediatric and Adolescent Diabetes clinical practice guidelines and the current guidelines were applied consistently to all patients throughout the study period.

### 2.4. Statistical Analysis

Data analysis was performed using SPSS (IBM, version 22). Quantitative data were described using measures of central tendency, such as mean and standard deviation (SD), or median and interquartile range, while frequencies and percentages were used to describe categorical data. Graphical representations, including histograms and box plots, were utilized for data visualization.

Differences between T1DM patients with and without CD were assessed using the Student’s *t*‐test for continuous variables and the chi‐square test or Fisher’s exact test for categorical variables. Statistical significance was set at *p* < 0.05. Correlation analysis of serological tests and duodenal biopsy in children and adolescents with diabetes and CD was performed using Pearson’s linear correlation. Binary logistic regression analysis was undertaken to identify predictors of CD in patients with T1DM. Variables included in the final model were those with significant *p*‐values in the binary logistic regression analysis.

### 2.5. Ethical Considerations

The study protocol was approved by the institutional review board (IRB) at King Abdullah International Medical Research Center under IRB number RJ18/083/J (approval date: 12 November 2018; extension approval date: 29 January 2020). All patient data were anonymized, and confidentiality was strictly maintained throughout the study. Informed consent was not needed for this study since it was a retrospective database study.

## 3. Results

The study enrolled 595 children and adolescents with T1DM receiving treatment and follow‐up at KAMC‐J (Figure 1), with 53.1% being males. The overall mean current age and mean age at diagnosis were 12.8 and 6.0 years, respectively. CD was diagnosed in 32 cases, yielding a prevalence rate of 5.5%. Among CD cases, 62.5% were females and 37.5% were males. No significant differences were noted in height SD score (height‐for‐age Z‐score) and weight SD score (weight‐for‐age Z‐score) between celiac and nonceliac cases. However, the mean systolic and diastolic blood pressures showed significant differences between children and adolescents with CD and without CD, with higher systolic blood pressure and lower diastolic blood pressure values in those with CD. The median HbA1c value among patients with CD (9.8%) was significantly higher than that for children and adolescents without CD (9.1%) (Table 1).

**FIGURE 1:**
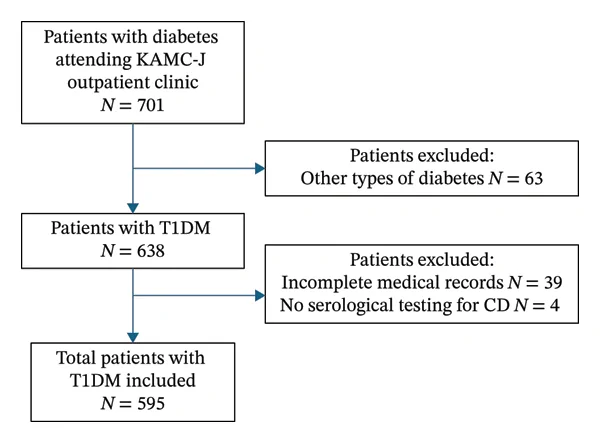
Study flowchart. Abbreviations: CD, celiac disease; KAMC‐J, King Abdulaziz Medical City‐Jeddah; T1DM, Type 1 diabetes mellitus.

**TABLE 1:** Basic characteristics of T1DM patients with and without CD.

Variable	Total	T1DM patients with CD	T1DM patients without CD	*p* value
*N* (%)	595	32 (5.4)	563 (94.6)	—
Gender				
Female	284 (47.7)	20 (62.5)	264 (46.9)	0.079^#^
Male	311 (52.3)	12 (37.5)	299 (53.1)	
Current age^∗^ (years)	12.8 ± 4.2	14.1 ± 3.7	13.1 ± 3.8	0.233
Age at diagnosis^∗^ (years)	6.0 ± 3.4	5.5 ± 3.8	6.0 ± 3.2	0.474
Height (SDS)^†^	0.31 (−0.41, 0.92)	0.20 (−0.54, 0.85)	0.44 (−0.22, 1.11)	0.311
Weight (SDS)^†^	0.55 (−0.10, 1.21)	0.48 (−0.08, 0.95)	0.58 (−0.06, 1.18)	0.545
SBP, mmHg^∗^	106.3 ± 19.6	100.4 ± 16.2	106.6 ± 19.8	0.040
DBP, mmHg^∗^	71.5 ± 15.7	78.7 ± 13.9	71.0 ± 15.7	0.006
HbA1c, %^†^	9.3 (8.2, 10.2)	9.8 (9.0, 11.0)	9.1 (8.1, 10.2)	0.005

*Note:* Values are *N* (%) unless specified. All *p*values are Fisher’s exact test *p* values, except where specified; HbA1c, hemoglobin A1c; IQR, interquartile range.

Abbreviations: CD, celiac disease; DBP, diastolic blood pressure; SBP, systolic blood pressure; SD, standard deviation; SDS, standard deviation score; T1DM, Type 1 diabetes mellitus.

^∗^Mean (± SD).

^†^Median (IQR).

^#^Chi square test *p* value.

Clinical and biochemical characteristics of children and adolescents with T1DM and CD versus children and adolescents without CD are presented in Table 2. Among those with T1DM and CD, 42.4% experienced frequent hypoglycemia episodes compared to 18.3% among children and adolescents without CD (*p* = 0.007). Furthermore, 25.0% of children and adolescents with T1DM and CD had three or more DKA episodes compared to 10.3% among children and adolescents without CD (*p* = 0.049). The prevalence of Hashimoto’s thyroiditis was significantly higher among those with CD (18.8%) compared to those without CD (8.3%) (*p* value 0.044). Total daily insulin dose per kilogram of body weight, severity of hypoglycemic episodes, presence of vitiligo, presence of adrenal insufficiency, and type of DM therapy did not differ between the two groups (*p* > 0.05) (Table 2). A significant difference in the type of insulin used was observed, with patients with CD using more short‐acting and less long‐acting insulin than patients without CD (*p* = 0.035).

**TABLE 2:** Clinical and biochemical data of patients with T1DM with and without CD.

Variable	Total	T1DM patients with CD	T1DM patients without CD	*p* value
*N* (%)	595	32 (5.4)	563 (94.6)	—
Type of DM therapy				
Intensive	581 (95.2)	32 (100.0)	549 (97.5)	0.361
Conventional	14 (2.3)	0 (0.0)	14 (2.5)	
Type of therapy				
MDI	570 (95.8)	32 (100.0)	538 (97.4)	0.357
Insulin pump	25 (4.2)	0 (0.0)	25 (4.5)	
Insulin type				
Short acting	349 (59.1)	22 (68.8)	326 (59.1)	0.035^#^
Long acting	242 (40.9)	10 (31.2)	226 (40.9)	
Total daily dose (u/kg/day)	1.1 ± 0.92	1.04 ± 0.33	1.09 ± 0.95	0.790
Hypoglycemia frequency				
Frequent^1^	117 (19.7)	14 (43.8)	103 (18.3)	0.007^#^
Infrequent^2^	137 (23.0)	4 (12.5)	133 (23.7)	
Rare^3^	145 (24.4)	7 (21.9)	138 (24.6)	
None	196 (32.9)	7 (21.2)	188 (33.4)	
Hypoglycemia severity				
Mild	365 (62.7)	10 (31.2)	341 (63.5)	0.213^#^
Moderate	31 (5.3)	11 (34.4)	30 (5.6)	
Severe	21 (3.6)	5 (15.6)	20 (3.7)	
None	165 (28.4)	6 (18.8)	146 (27.2)	
DKA				
None	401 (67.4)	19 (59.4)	375 (67.9)	0.049^#^
1 episode	95 (20.0)	3 (9.4)	90 (16.3)	
2 episodes	33 (5.5)	2 (6.3)	30 (5.4)	
3+ episodes	66 (11.1)	8 (25.0)	57 (10.3)	
Hashimoto’s thyroiditis				
Yes	54 (9.1)	6 (18.8)	46 (8.3)	0.044^#^
No	541 (90.9)	26 (81.2)	506 (91.7)	
Vitiligo				
Yes	6 (1.0)	0 (0)	6 (1.1)	0.553
No	589 (99.0)	32 (100)	557 (98.9)	
Adrenal insufficiency				
Yes	2 (0.3)	0 (0)	2 (0.4)	0.733
No	593 (99.7)	32 (100)	561 (99.6)	

*Note:* All *p* values are Fisher’s exact test *p* values, except where specified; DKA, diabetic ketoacidosis.

Abbreviations: CD, celiac disease; DM, diabetes mellitus; MDI, multiple daily injection; T1DM, Type 1 diabetes mellitus.

^1^3 episodes in the last 3 visits.

^2^2 episodes in the last 3 visits.

^3^1 episode in the last 3 visits.

^#^Chi square test *p* value.

Celiac screening results are summarized in Table 3, with all tests revealing significant associations (*p* values < 0.05). The TTG test, at diagnosis and follow‐up, exhibited the higher sensitivity and specificity compared to EMA and AGA tests. The AGA test demonstrated the lowest sensitivity and specificity for CD screening (Table 3). The TTG test at diagnosis demonstrated a high correlation with duodenal biopsy in children and adolescents with diabetes and CD (correlation coefficient = 0.78).

**TABLE 3:** Celiac screening results in T1DM patients with CD compared to T1DM patients without CD.

Variable	Total	T1DM patients with CD	T1DM patients without CD	Sensitivity	Specificity	*p* value
*N* (%)	595	32 (5.4)	563 (94.6)	—	—	—
TTG at diagnosis						
Positive	56 (9.4)	20 (62.5)	36 (6.5)	64.5	91.0	0.001
Negative	375 (63.0)	11 (34.4)	364 (65.9)			
Not performed	164 (27.6)	1 (3.1)	163 (29.6)			
TTG at follow‐up						
Positive	40 (6.7)	26 (81.3)	14 (2.5)	92.8	93.2	0.001
Negative	195 (32.8)	2 (6.2)	193 (34.3)			
Not performed	360 (60.5)	4 (12.5)	356 (63.2)			
EMA at diagnosis						
Positive	53 (8.9)	21 (65.6)	32 (5.7)	39.6	89.5	0.001
Negative	190 (31.9)	6 (18.8)	170 (30.2)			
Not performed	352 (59.2)	5 (15.6)	361 (64.1)			
TTG or EMA at diagnosis						
Positive	109 (18.3)	21 (65.7)	88 (15.6)	65.7	81.6	0.001
Negative	402 (67.6)	11 (34.4)	391 (69.5)			
Not performed	84 (14.1)	0 (0)	84 (14.9)			
AGA at diagnosis						
Positive	22 (3.7)	15 (46.9)	7 (1.3)	68.2	71.0	0.001
Negative	168 (28.2)	4 (12.5)	164 (29.1)			
Not performed	405 (68.1)	13 (40.6)	392 (69.6)			
Duodenal biopsy						
Positive	32 (6.2)	32 (100)	0 (0)	100	100	0.001^#^
Negative	12 (2.0)	0 (6.2)	12 (2.1)			
Not performed	546 (91.7)	0 (0)	551 (97.9)			

*Note:* All *p* values are chi square *p* values, except where specified; EMA, endomysial antibody.

Abbreviations: AGA, anti‐gliadin antibodies; CD, celiac disease; T1DM, Type 1 diabetes mellitus; TTG, tissue transglutaminase.

^#^Fisher’s exact test *p* value.

Celiac patients exhibited a higher prevalence of vitamin D deficiency (25‐OH‐Vitamin *D*3  < 20 ng/dL) (84.4%) compared to nonceliac patients (57.7%) (*p* = 0.003). The proportion of patients with a HbA1c more than 8% was higher among celiac cases (78.1%) compared to the nonceliac group (69.1%) (*p* = 0.011) (Table 4). The distribution of glycemic control among T1DM with and without CD is illustrated in Figure 2.

**TABLE 4:** Vitamin D status and HbA1c level in T1DM patients with and without CD.

Variable	Total	T1DM patients with CD	T1DM patients without CD	*p* value
*N* (%)	595	32 (5.4)	563 (94.6)	—
25‐OH‐Vitamin D, ng/dL				
Deficient (< 20 ng/dL)	359 (60.3)	27 (84.4)	325 (57.7)	0.003
Normal	236 (39.7)	5 (15.6)	238 (42.3)	
HbA1c, %				
8% and less	172 (30.3)	7 (21.9)	165 (30.9)	0.011
More than 8%	395 (69.7)	25 (78.1)	370 (69.1)	

*Note:* All *p* values are chi square test *p* values; HbA1c, hemoglobin A1c.

Abbreviations: CD, celiac disease; T1DM, Type 1 diabetes mellitus.

**FIGURE 2:**
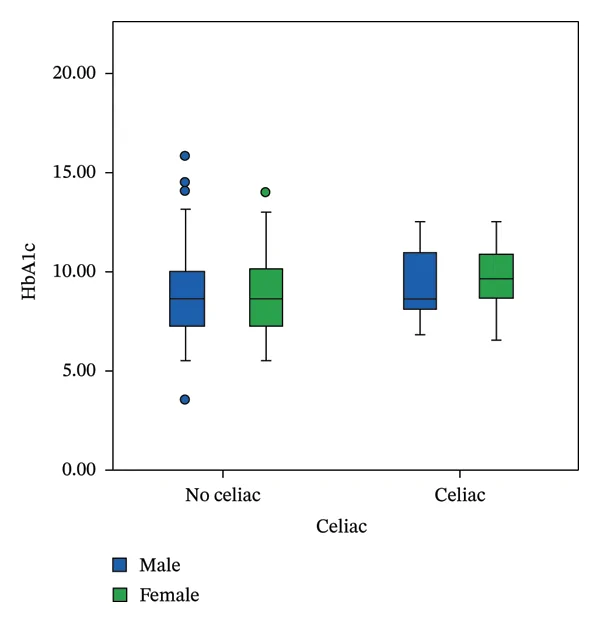
Distribution of glycemic control among T1DM patients with and without CD. Abbreviations: CD, celiac disease; HbA1c, hemoglobin A1c; T1DM, Type 1 diabetes mellitus.

The results of the binary logistic regression model reported significant impact of HbA1c and duodenal biopsy on CD prevalence (Table 5). Children and adolescents with higher HbA1c values and positive duodenal biopsy were more likely to develop CD controlling for other factors included in the model.

**TABLE 5:** Binary logistic regression of CD predictors.

Variable	Or (95% CI)	*p* value
Gender		
Female (ref)	—	—
Male	0.81 (0.28, 2.30)	0.698
Current age (years)	1.01 (0.95, 1.07)	0.880
Height	0.99 (0.95, 1.04)	0.788
Weight	1.01 (0.97, 1.06)	0.606
HbA1c	1.35 (1.02, 1.83)	0.049
Insulin type		
Short acting (ref)	—	—
Long acting	0.52 (0.18, 1.44)	0.211
Hypoglycemia frequency		
Frequent	0.47 (0.05, 4.66)	0.518
Infrequent	2.16 (0.57, 8.19)	0.254
Rare	1.93 (0.51, 7.32)	0.336
None (ref)	—	—
DKA		
None (ref)	—	—
1 episode	0.51 (0.10, 2.57)	0.416
2 episodes	0.87 (0.09, 8.16)	0.906
3+ episodes	1.68 (0.37, 7.67)	0.502
TTG at diagnosis		
Positive	0.05 (0.01, 1.34)	0.996
Negative	1.71 (0.52, 5.76)	0.387
Not performed (ref)	—	—
TTG at follow‐up		
Positive	0.55 (0.05, 1.45)	0.998
Negative	0.33 (0.09, 1.17)	0.087
Not performed (ref)	—	—
EMA at diagnosis		
Positive	0.46 (0.07, 2.85)	0.407
Negative	1.35 (0.38, 4.71)	0.639
Not performed (ref)	—	—
AGA at diagnosis		
Positive	0.04 (0.01, 1.55)	0.998
Negative	0.34 (0.07, 1.52)	0.160
Not performed (ref)	—	—
Duodenal biopsy		
Positive	5.22 (1.29, 17.6)	0.030
Negative	5.87 (0.65, 5.71)	0.114
Not performed (ref)	—	—

*Note:* DKA, diabetic ketoacidosis; EMA, endomysial antibody; HbA1c, hemoglobin A1c; TTG, tissue transglutaminase.

Abbreviations: AGA, anti‐gliadin antibodies; CD, celiac disease; CI, confidence interval; OR, odds ratio.

## 4. Discussion

This study has highlighted a high prevalence of CD in children and adolescents with T1DM from Saudi Arabia, with positive CD serological tests in 9.4% and a positive biopsy in 5.4% of participants. Duodenal biopsy confirmed CD in 62.5% of children and adolescents with positive TTG, 65.6% with positive EMA, and 46.9% with positive AGA. The TTG test at diagnosis demonstrated the highest specificity (91.0%), while the AGA test exhibited the highest sensitivity (68.2%).

Patients with T1DM face an elevated risk of developing autoimmune disorders, including CD, Hashimoto’s thyroiditis, Addison’s disease, atrophic gastritis, pernicious anemia, and vitiligo [16, 17]. The association between T1DM and CD has been recognized since 1969, primarily due to shared genetic risk factors involving HLA genes, particularly HLA‐DQ2 and HLA‐DQ8, which increase susceptibility to CD in patients with T1DM [28]. Recent studies have reinforced this genetic link, emphasizing the importance of genetic screening in at‐risk populations [29].

Globally, the prevalence of CD among children with T1DM varies widely. Studies have reported serological test prevalence rates for CD ranging from 1.4% to 24.5% in children with T1DM [9]. In Western countries, CD affects approximately 1%–2% of the general population, while the prevalence among those with T1DM is notably higher, at 2%–12% [30]. Based on intestinal biopsy, global CD prevalence in children with T1DM ranges from 1.6% to 16.4% [31]. The serological prevalence of CD 9.4% in this study far exceeds the 3.2% prevalence of CD in children without diabetes in Saudi Arabia [32]. Recent studies from Saudi Arabia have reported similar high prevalence of CD, between 11.5% and 21.2% [10–15]. Such high prevalence rates underscore the need for routine screening in this high‐risk group.

Recent advancements in serological testing have further improved diagnostic accuracy, allowing for earlier intervention and management in patients with T1DM and CD [33]. Our findings suggest that the TTG test at diagnosis has the highest specificity, while the AGA test has the highest sensitivity. Badizadegan et al. found a 100% probability of histologically confirmed CD in children with TTG values > 2000 Chem’U, emphasizing the diagnostic utility of high TTG levels [34]. Kaur et al. reported that highly positive serology (TTG titer > 200 RU/mL) with clinical symptoms increased specificity to 100%, potentially eliminating the need for endoscopy and pathology in some cases [35]. A meta‐analysis study, including 113 studies, reported sensitivity and specificity of serological assays in children; TTG showed 97.7% sensitivity and 70.2% specificity while EMA showed 94.5% sensitivity and 93.8% specificity [36].

Clinical practice guidelines from the International Society for Pediatric and Adolescent Diabetes and the American Diabetes Association recommend CD screening at the time of T1DM diagnosis, then 2 and 5 years afterward, or sooner if symptoms arise. More frequent screening is advised for children with a first‐degree relative who has CD [37, 38]. Either TTG or EMA are recommended for screening purposes [37, 38]. Managing both T1DM and CD can complicate diabetes management, especially with regard to nutrition. Gluten‐induced gut inflammation affects nutrient absorption, and GF alternatives often contain more carbohydrates than gluten‐containing foods, potentially impacting glycemic control. A well‐managed GF diet can enhance nutrient absorption, maintain a healthy weight, and stabilize blood sugar levels [39–41]. Recent dietary studies suggest that individualized nutrition plans tailored to the needs of patients with T1DM and CD can improve long‐term health outcomes [16].

In this study, the prevalence of autoimmune hypothyroidism was higher in children and adolescents with both T1DM and CD (18.8%) compared to those with only T1DM (8.3%). Similarly, a nationwide cohort study in Sweden indicated CD as a significant risk factor for hypothyroidism in T1DM patients, with a hazard ratio of 1.67 (95% confidence interval 1.32, 2.11) [42]. Additionally, recent evidence suggests that thyroid dysfunction may complicate glycemic control and increase cardiovascular risk in patients with T1DM and CD [43]. However, another study suggested that Hashimoto’s thyroiditis did not significantly impact T1DM control or its complications, highlighting the need for further research on the complex interplay between these conditions [44].

In this study, the linear growth and body mass index (BMI) of T1DM patients with CD did not differ from those without CD, suggesting normal growth patterns. This may be due to the asymptomatic or mild nature of CD symptoms in most patients or adherence to a GF diet. Studies have shown varied results with regard to anthropometric measurements in patients with T1DM and CD. Berioli et al. demonstrated that mean growth was similar between children with T1DM and CD and those with only T1DM treated with a GF diet for 1 year [45]. Similarly, Barera et al. showed no difference in BMI between children with T1DM who developed CD compared to those who did not; however, BMI improved following the introduction of a GF diet [46]. On the other hand, a cohort study including 19,796 children with T1DM demonstrated that SD scores for BMI, weight, and height were significantly less in children with T1DM and CD compared to those without CD [47]. Proper dietary management is crucial in patients with T1DM in order to achieve normal blood glucose levels, blood pressure, and lipid profiles, and a healthy body weight. Dietary advice should focus on carbohydrate quantity, type, and distribution, emphasizing low glycemic index foods [48, 49]. Regular monitoring and adjustments of dietary intake based on glycemic responses could improve metabolic control in these patients.

Studies have shown inconsistent results regarding the influence of a GF diet on glycemic control, insulin dosage, HbA1c, glucose excretion, and hypoglycemic episodes in patients with both T1DM and CD [25, 50, 51]. This study revealed significantly higher HbA1c levels and more frequent episodes of DKA in T1DM patients with CD compared to those without CD. Taler et al. showed similar HbA1c as well as frequency of DKA and severe hypoglycemic events in T1DM patients with CD treated with a GF diet compared to those not treated with a GF diet [50]. Hegab et al. demonstrated that patients with T1DM and CD with villous atrophy had significantly higher HbA1c levels compared to patients with only T1DM [52]. A recent Norwegian study comparing macronutrient content in GF and gluten‐containing products found higher carbohydrate, saturated fat, and salt content but lower protein and fiber in GF products [53]. Another review identified key inadequacies in GF products, such as low protein and high fat and salt content [54]. Diabetic patients often pursue a low glycemic index diet to manage glucose excursions, which are linked to increased long‐term microvascular complications [55]. The consumption of GF foods with a high glycemic index results in more rapid and higher rises in blood glucose levels, partially explaining the higher HbA1c in T1DM patients with CD versus those without CD [56].

Conversely, uncontrolled CD, characterized by gluten ingestion, causes intestinal mucosal inflammation, impairing food absorption and potentially causing hypoglycemia. Some GF alternatives are made with low‐carbohydrate substitutes, leading to hypoglycemia after standard insulin dosages [26]. In contrast to our findings, a Swedish cohort study found no influence of CD on the risk of coma, ketoacidosis, or hypoglycemia in patients with T1DM [21]. Recent insights suggest that improved education and adherence to dietary recommendations can mitigate these risks [57].

The study used a large cohort of 595 Saudi children and adolescents with T1DM, providing robust data on the prevalence of CD and other autoimmune disorders. Both serological and biopsy methods were employed for accurate CD diagnosis, enhancing the reliability of our findings. This research offers practical insights into the clinical management of T1DM with coexisting autoimmune conditions, supporting current guidelines. Despite the strengths of the study, several limitations exist. This retrospective study was limited by the lack of data on patient symptoms, nutrient uptake, adherence to a GF diet, and family history of CD, which prevented a comprehensive evaluation of these factors. Additionally, the absence of longitudinal data and reliance on single‐point measurements restricts our ability to infer causality or assess progression of disease over time. Furthermore, the high number of patients without CD for whom serological tests were “not performed” is a potential limitation; however, the combined TTG or EMA at diagnosis variable demonstrates only 14.1% of patients did not have TTG or EMA serological testing at diagnosis. Additionally, diagnostic challenges must also be mentioned, including dietary changes, illness, and IgA deficiency, which can affect serology results [58]. Future research should include additional data concerning the timing of CD diagnosis, dietary patterns, treatment variations, lifestyle difference, or presence of subtle autoimmune features to further enhance the analysis as well as to optimize hypoglycemia management and glycemic control in patients with both T1DM and CD. We recommend routine screening for CD using TTG tests in children and adolescents with T1DM, for early detection and management as well as the development of integrated care plans that include personalized dietary management and monitoring of vitamin D levels.

## 5. Conclusions

This study has provided insights into the high prevalence of CD in children and adolescents with T1DM from Saudi Arabia, with patients with CD experiencing higher HbA1c and Hashimoto’s thyroiditis than those without CD. Celiac screening results demonstrated that the TTG test had the highest sensitivity and specificity at diagnosis and follow‐up, compared to EMA and AGA tests. The results highlight that a precise diagnosis of CD using serological testing and selected endoscopic evaluation, and dietary control alongside prandial insulin dose adjustments, are necessary for achieving glycemic control in these patients.

## Author Contributions

Adnan AlShaikh proposed the research, set the study design, supervised the study, and wrote the manuscript. Mohamed Ahmed statistically analyzed the data and wrote the results section. Abdulaziz Alghamdi, Sahl Alshaikh, Yahya Almatihmy, Aljowharah AlSehaibany, Leena Waqar, Joud Alshaikh, and Shaima Alghamdi helped with the study design, data collection, and clearance process. Mohammed Hasosah and Ashraf Soliman helped with the sections of results, discussion, and referencing.

## Funding

There was no funding related to the present study.

## Disclosure

All authors have read and approved the final form of the manuscript.

## Ethics Statement

Ethical approval was granted from King Abdullah International Medical Research Center, Jeddah, Saudi Arabia (IRB approval no.: RJ18/083/J; approval date: 12 November 2018; extension approval date: 29 January 2020). Consent was not needed for this study since it was a retrospective database study.

## Conflicts of Interest

The authors declare no conflicts of interest.

## Supporting Information

Additional supporting information can be found online in the Supporting Information section.

## Supporting information


**Supporting Information** Supporting File Appendix 1**.** Technical Specifications for the Celiac Disease Serological Assays.

## Data Availability

The datasets used and/or analyzed during the present study are available from the corresponding author upon reasonable request.
