# Supplementary material for: Prevalence of Celiac Disease in Children and Adolescents With Type 1 Diabetes in Saudi Arabia: A 20‐Year Experience in a Tertiary Care Center
Source: Int J Endocrinol. 2026 Nov 9;2026:5921264. doi: 10.1155/ije/5921264 (PMC13748945; doi:10.1155/ije/5921264)
Supplement: Supplementary file 1 [file IJE-2026-5921264-s001.docx]

**Prevalence of Celiac Disease in Children with Type 1 Diabetes in Saudi Arabia: A 20-year Experience in a Tertiary Care Center**

Supplementary File 1

**Appendix 1.** Technical Specifications for the Celiac Disease Serological Assays

| **Test Name** | **IgA Autoantibodies Against Tissue Transglutaminase (Anti-tTG IgA)** |
| --- | --- |
| Clinical Use | Diagnosis of Celiac Disease |
| Method / Principle | Enzyme-Linked Immunosorbent Assay |
| Sample Type | Serum |
| Measuring Range (AMR) | 1.23–100 U/mL |
| Result Interpretation | Negative: <4 U/mL  Weak Positive: 4–10 U/mL  Positive: >10 U/mL |
| Precision (Within-run / Intra-assay) | CV ≤5.5% |
| Precision (Between-run / Inter-assay) | CV ≤7.1% |
| Relative Sensitivity | 98.2% |
| Relative Specificity | 94.2% |

| **Test Name** | **Gliadin IgA Antibodies** |
| --- | --- |
| Clinical Use | Semi-quantitative detection of IgA antibodies to gliadin to aid in the evaluation of celiac disease |
| Method / Principle | Enzyme-Linked Immunosorbent Assay |
| Sample Type | Serum |
| Cut-off Value | 20 U |
| Result Interpretation | Negative: <20 U  Weakly Positive: 20.8–25.8 U  Positive: ≥35 U |
| Within-run (Intra-assay) Precision | CV ≤8.5% |
| Between-run (Inter-assay) Precision | CV ≤16.4% |

| **Test Name** | **Gliadin IgG Antibodies** |
| --- | --- |
| Clinical Use | Detection of IgG antibodies to gliadin to aid in the evaluation of celiac disease, particularly in patients with suspected gluten-sensitive enteropathy |
| Method / Principle | Enzyme-Linked Immunosorbent Assay |
| Sample Type | Serum |
| Result Interpretation | Negative: <20 U  Weak Positive: 20–30 U  Moderate to Strong Positive: >30 U |
| Cut-off Value | 20 U |
| Within-run (Intra-assay) Precision | CV ≤10.0 |
| Between-run (Inter-assay) Precision | CV ≤9.8% |

| **Test Name** | **EMA** |
| --- | --- |
| Clinical Use | Screening for IgA-class autoantibodies against smooth muscle endomysium to aid in the diagnosis of coeliac disease. |
| Method / Principle | Indirect immunofluorescence assay using primate tissue substrates |
| Sample Type | Serum |
| Result Interpretation | Negative: < 1:10 |
| Units | Titer |
| Turnaround Time | 15 days |
| Kit Provider | IMMCO Diagnostics |
| Specimen Quantity | 1ml blood (for pediatric patients) |
